# Glide Path in Endodontics: A Literature Review of Current Knowledge

**DOI:** 10.3390/dj12080257

**Published:** 2024-08-14

**Authors:** Vlad Mircea Lup, Giulia Malvicini, Carlo Gaeta, Simone Grandini, Gabriela Ciavoi

**Affiliations:** 1 Doctoral School of Biomedical Sciences, University of Oradea, 410 087 Oradea, Romania; dr.vladlup@yahoo.com; 2 Unit of Endodontics and Restorative Dentistry, Department of Medical Biotechnologies, University of Siena, 53100 Siena, Italy; giulia.malvicini@student.unisi.it (G.M.); c.gaeta@unisi.it (C.G.); 3 Faculty of Medicine and Pharmacy, University of Oradea, 410 087 Oradea, Romania; gciavoi@uoradea.ro

**Keywords:** glide path, reciprocation, root canal treatment, rotary, shaping

## Abstract

The introduction of nickel–titanium rotary instruments revolutionized shaping procedures as they were able to produce a well-tapered preparation while reducing operator fatigue. The major drawback of rotary instruments was the high risk of fracture due to bending and torsional stress. Thus, the creation of a glide path has been advocated and recommended by most rotary instrument manufacturers. The aim of the present review is to summarize existing knowledge on glide path preparation and identify areas where further research is needed. The primary goal is to provide a comprehensive overview of the techniques and instruments used in glide path preparation, highlighting their advantages and limitations. The secondary goal is to explore the effect of glide path creation on the overall success of endodontic treatment, particularly in terms of reducing procedural errors and improving treatment outcomes. An online search on PubMed, ScienceDirect, UCLA, and Scopus databases was conducted, and 116 articles were identified. Eligible articles were divided into nine categories based on what they researched and compared. The categories included centering ability and/or root canal transportation, cyclic fatigue resistance, glide path and shaping time, tortional stress resistance, apical extrusion of debris and/or bacteria, defects in dentine walls, file separation, postoperative pain assessment, and scouting ability and performance. Establishing a glide path reduces root canal transportation, especially with rotary methods. Reciprocating and heat-treated files offer higher fatigue resistance and shorter preparation time. Instruments with shorter pitch lengths have greater torsional strength. Preparation and coronal preflaring reduce apical debris and bacteria. Glide paths do not affect dentine microcracks, file separation, or defects but reduce immediate postoperative pain and improve cutting ability. Randomized trials are needed to assess their impact on treatment outcomes.

## 1. Introduction

Root canal treatments involve three major phases—shaping, cleaning, and obturation. Although they are strongly interconnected, the shaping of the root canal system is often regarded as the most important step in endodontic therapy because it influences the efficiency of the cleaning and obturation steps.

In 1974, Schilder changed the way we perceived endodontic protocols with his concepts that shed light on the mechanical and biological principles needed to achieve an optimal canal shape [1]. He proposed several objectives: ensuring that the shaped root canal has a smooth, flowing taper from the orifice to the terminus; keeping the foramen as small as practical and in its original position; making sure that the prepared shape follows the original anatomy of the root canal; limiting the preparation to only the root canal space; allowing the removal of tissue from inside the root canal without forcing debris over the foramen; and creating a final shape that permits the placement of various medicaments and the proper exchange of irrigants.

The first steps in root canal instrumentation are canal scouting and preflaring the canal. They are regarded as the most challenging and controversial phases in root canal shaping. The main issues include [2] locating, accessing, and enlarging the main canals without procedural errors; maintaining a proper working length throughout the shaping procedures; and selecting the size and geometry of the shape to facilitate efficient disinfection and obturation.

A series of technical protocols have been developed to ensure the achievement of Schilder’s objectives and to minimize the percentage of procedural errors. That is when serial instrumentation was implemented using multiple hand files and reamers, and a series of techniques were developed for shaping the root canal space [1].

The step-back technique was focused on preparing the apical portion of the canal first, and only then the coronal flaring [3]. The crown-down technique began the preparation with large files at the orifice and in the coronal portion of the canal, followed by progressively smaller files as the preparation advanced to the middle and apical thirds [4]. Roane’s balanced force technique permitted the shaping of curved canals using a combined series of movements [5]. But most of the problems encountered in shaping curved root canals were due to the stiffness of stainless steel instruments, and their tendency to straighten inside the canal, thus resulting in an uneven force distribution in outer curves or convexities of the canal [6].

The introduction of nickel–titanium (Ni-Ti) rotary instruments in the mid-90s revolutionized the shaping procedures due to their lower module of elasticity and thus exertion of smaller forces on the dentine walls in curved canals [7]. One of the main advantages of rotary Ni-Ti files was that they were able to produce a good well-taper shape while reducing operator fatigue [8], but the taper lock effect might occur in narrow canals [9] leading to several procedural errors such as ledge formation, perforation, or even file separation [10].

Even with their major advantages over stainless steel files, the major drawback of rotary Ni-Ti instruments was the high fracture risk [11] due to cyclic flexural fatigue (also known as bending stress) or through shear stress (also known as torsional stress) [12]. Torsional stress occurs when there is a big contact area between the instrument’s cutting blade and the dentinal walls [13], the cross-section of the root canal is narrower than the cross-section of the instrument’s tip [13], or an excessive apical pressure on the handpiece is exerted during the shaping procedures [14].

To reduce torsional stress and counter the taper lock effect that might occur due to the non-cutting tip of the rotary instrument, the creation of a glide path has been advocated [15].

By definition, the glide path is a smooth, reproductible passage from the root canal’s orifice to its physiological terminus [16]. Nowadays, most rotary Ni-Ti instrument manufacturers recommend the creation of a glide path before rotary canal shaping in order to remove coronal interferences [17], respect the original canal anatomy [18], and reduce the incidence of procedural errors and apical extrusion of debris [19].

At first, the glide path was created manually with the use of small stainless steel K-files as it was a reliable technique [20]. West formulated that a glide path is present when a size 10 K-file can move freely inside the root canal [21], Van der Vyver considered a safe glide path is when a size 15 K-file can slide easily to the working length without any rotation applied to it [22], and Bergmans stated that no rotary Ni-Ti instrument should go where a hand instrument has been before [23]. However, creating a manual glide path can often be time-consuming [9], technique sensitive [17], and may lead to poor outcomes [24].

Therefore, in conjunction with metallurgical advancements, specialized rotary Ni-Ti instruments were developed for glide path preparation. Initially, series of glide path instruments, such as the PathFile system (Dentsply Meillefer, Ballaigues, Switzerland), were introduced. These were followed by single-file instruments designed for both continuous rotation, like the ProGlider (Dentsply Sirona, Ballaigues, Switzerland), and reciprocation motion, such as the R-Pilot (VDW, Munich, Germany) and the WaveOne Gold Glider (Dentsply Sirona, Ballaigues, Switzerland).

Numerous studies have been conducted to elucidate the concept of glide path preparation and evaluate the advantages of different rotary Ni-Ti instruments used for this purpose. The aim of the present review is to summarize existing knowledge on glide path preparation and identify areas where further research is needed. The primary goal is to provide a comprehensive overview of the techniques and instruments used in glide path preparation, highlighting their advantages and limitations. The secondary goal is to explore the impact of glide path creation on the overall success of endodontic treatment, particularly in terms of reducing procedural errors and improving treatment outcomes.

## 2. Literature Search

This review was performed according to the recommendations of the PRISMA (Preferred Reporting Items for Systematic Review and Meta-Analysis) statement [25].

For this review, a search that ended in December 2023 was conducted in PubMed, Science Direct, UCLA and Scopus databases.

**Figure 1:**
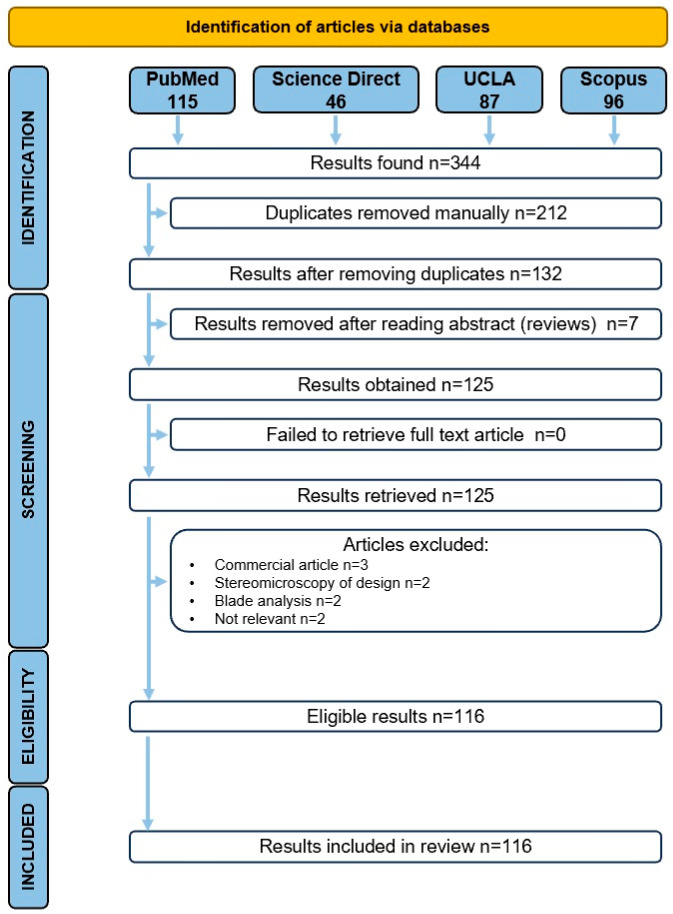
Flowchart following the PRISMA principles showing the database search and the selection processes.

The search criteria included articles in English, within the dentistry field, published from the year 2000 to the present, presenting “glide path” in their title, limiting the search strategy to “glide path [tab]” to include all the relevant articles. A preliminary search yielded 344 articles (PubMed 115, Science Direct 46, UCLA 87, Scopus 96). Duplicates were manually selected and discarded with records kept for reference. After removing duplicates, 132 unique articles remained. Upon further screening of their title and abstract, 7 more articles were excluded as they were review articles, so 125 articles remained. A meticulous examination of the abstracts led to the exclusion of 9 additional articles that were deemed irrelevant to the review’s focus (e.g., commercial articles, blade analysis of instruments, stereomicroscopy of design), leaving 116 articles. A thorough search of each article’s references was conducted to check if any new article could be included in this review. As all eligible papers found were already included, no extra articles were added after this step. Figure 1 presents the search process, identification, inclusion and exclusion phase.

Each article included in this review was retrieved, thoroughly read, and categorized according to the topics investigated. In cases where an article addressed multiple topics, it was indexed under all relevant categories.

## 3. Discussion

After reading all the articles included in the present review, we identified nine distinct categories, based on the topics they researched, analyzed, or compared.

### 3.1. Centering Ability and/or Root Canal Transportation

This was unequivocally the most extensively researched topic concerning the glide path (*n* = 40 articles). Within this category, two major sub-topics were identified: analyzing the centering ability and root canal transportation of two or more glide path rotary Ni-Ti files and examining the centering ability and root canal transportation of shaping files after glide path was established.

#### 3.1.1. Comparison of the Centering Ability and Root Canal Transportation of Two or More Glide Path Rotary Ni-Ti Files

A total of 19 articles addressed this sub-topic, as they compared different glide path rotary Ni-Ti files among each other or compared them with manual files for glide path preparation. Articles, methods for evaluation, and results are shown in Table 1.

#### 3.1.2. Analyzing the Centering Ability and Root Canal Transportation of Shaping Files after Glide Path Was Established

A total of 21 studies addressed this subtopic as they compared canal transportation of different shaping files after establishing a glide path with different glide path rotary Ni-Ti files. This subtopic can be further divided into three distinct categories:

- Analyzing canal transportation of different shaping files with or without glide path established (*n* = 8);
- Comparing canal transportation of different shaping files after glide path was established (*n* = 8);
- Analyzing canal transportation of a shaping file after different glide path files were used (*n* = 5).

Articles, methods for evaluation, and results for the first category are shown in Table 2.

In the second category, articles compared canal transportation of different shaping files after a glide path was established. Articles, methods for evaluation, and conclusions are shown in Table 3.

In the third category, articles analyzed the centering ability and canal transportation of a single shaping file after glide path was established with different files. Articles, methods for evaluation, and conclusions can be found in Table 4.

#### 3.1.3. Topic Conclusions

Only two articles [17,28] found no significant difference in apical transportation and centering ability when comparing rotary glide path files with stainless steel hand K-files. Both studies used superimposed X-rays to assess the results. Conversely, all other studies in this category concluded that rotary glide path files caused less canal transportation and stayed more centered in the root canal, thus respecting the original canal anatomy. Notably, among continuous rotation glide path files, Pathfiles and the ProGlider file demonstrated superior outcomes. Additionally, no significant differences were found among continuous rotation files when used in optimized glide path motion. In studies comparing reciprocating files with continuous rotation files, the WaveOne Gold Glider (Dentsply Sirona, Ballaigues, Switzerland) and the R-pilot (WDV, Munich, Germany) caused less canal transportation and showed better respect for the original root canal anatomy. It would benefit all clinicians if new studies were conducted promptly upon the introduction of other glide path files to the market, such as the ProTaper Ultimate Slider (Dentsply Sirona, Ballaigues, Switzerland). Additionally, further research into reciprocating glide path files, which appear to offer superiority in this area, would be valuable.

When comparing the centering ability and canal transportation of a shaping file with or without a prior glidepath established, most studies conclude that an established glide path before the use of shaping files greatly reduce transportation regardless of the type of motion of the shaping file (continuous rotation or reciprocation). Only [43,46] found that, of the shaping files studied (MTwo and ProTaper Universal, respectively), glide path had no influence in apical transportation. Both of them used superimposed X-rays as a method for concluding the results.

Articles comparing different shaping files after a glide path was established suggest that reciprocating shaping files tend to induce less transportation and maintain better centering within the root canal.

Also, shaping files perform significantly better in terms of transportation and centering when a glide path is established, with minor differences between the types of glide path files used.

### 3.2. Cyclic Fatigue Resistance

The second topic addressed when talking about glide path is the cyclic fatigue resistance of different glide path files (*n* = 23 articles). Cyclic fatigue resistance can be measured in a static or dynamic mode, usually in a stainless-steel block with a milled simulated canal of 45°, 60° or 90° curvature with the radius of that curve typically being 3 or 5 mm. The results compared when talking about cyclic fatigue are the time to fracture (TTF) and the number of cycles to fracture (NCF).

Some articles compared the cyclic fatigue resistance of different shaping files with a control group that used brand new files and other groups with those files being tested after they were used with or without a glide path.

- In 2018, Özyürek et al. [63] compared the cyclic fatigue resistance of Reciproc (*n* = 60) and Reciproc Blue (*n* = 60) files in a simulated canal with a 60° curvature and a radius of 5 mm. Each group had new files (*n* = 20), files that prepared three root canals of mandibular molars without a glide path (*n* = 20), and files that prepared three root canals of mandibular molars with a previous glide path achieved with ProGlider (*n* = 20). The NCF was significantly higher for Reciproc Blue in all groups tested.
- In 2019, Uslu and Inan [64] compared the cyclic fatigue resistance of new WaveOne Primary files (*n* = 10) with files that previously prepared a J-shape acrylic block with no glide path (*n* = 10) or with a glide path established by Profile (*n* = 10) or ProGlider files (*n* = 10). The cyclic fatigue tests were conducted in a simulated canal with a 60° curvature and a radius of 5 mm and the NCF was recorded. New WaveOne files had significantly greater NCFs (741.36 ± 71.52) than the other three groups with a significant difference between the ProGlider + WaveOne group and the other two groups (668.18 ± 79.10 vs. 644.42 ± 81.97 and 605.28 ± 66.75).
- In 2020, Ates et al. [65] compared the cyclic fatigue resistance of the new XP Shapers (*n* = 32) at 1000 and 3000 rpm with those used for shaping four 3D printed root canals without a glide path (*n* = 32) or with a glide path established by #10, #15 and #20 K-files (*n* = 32). The cyclic fatigue tests were carried out in a simulated canal with a 75° curvature and a radius of 7.5 mm and the NCF and TTF were recorded. The 3000 rpm groups had higher NCFs (*p* < 0.05), and the 1000 rpm groups higher TTFs. No statistical difference was found between the groups regardless of whether the files were used at 1000 rpm or 3000 rpm with or without a glide path (*p* > 0.05).
- In 2023, Scherer et al. [66] compared the cyclic fatigue resistance of new WaveOne Gold Primary files (*n* = 6) with those used to prepare a mandibular molar with a glidepath established by K-files (*n* = 6) or WaveOne Gold Glider (*n* = 6). The cyclic fatigue tests were conducted in a simulated canal with a 60° curvature and a radius of 5 mm and the NCFs were recorded. No significant differences were found between groups; thus, they concluded that creating a glide path does not affect the cyclic fatigue resistance of reciprocating instruments.

One article by Kwak et al. [67] used prototype files with two different pitch lengths, 0.14 mm at the tip and 3% taper, with a heat treatment applied or not. Cyclic fatigue tests were conducted at 300 rpm in a simulated canal with a length of 17 mm, a 90° curvature and a radius of 3 mm, and the NCFs were recorded. An increase in the resistance to cyclic fatigue was observed in the heat-treated groups in relation to non-heat-treated groups, and in the short pitch groups in relation to the long pitch ones (*p* < 0.05). The rest of the articles on this topic, which compared the cyclic fatigue of different glide path files, can be consulted in Table 5 along with their method of determination and results.

Based on the findings from articles on this topic, reciprocating glide path files and heat-treated glide path files typically exhibit higher cyclic fatigue resistance. It would greatly benefit clinicians if more studies were conducted to determine cyclic fatigue resistance in dynamic mode, thus providing a closer simulation of handpiece movement.

### 3.3. Glide Path and Shaping Time

Articles on this topic (*n* = 14) analyzed the time certain files needed to work inside the canal and can be divided as follows:

- The time needed to perform a glide path with rotary glide path files versus manual files;
- The time needed to establish a glide path with different rotary glide path files;
- The time of different shaping files to reach working length after different glide paths.

Paleker et al. in 2017 [86] and D’Amario et al. in 2013 [28] both compared the glide path time of G-files (G1 and G2), k-files (#10, #15 and #20) with ProGlider and Pathfile (PF 1, 2 and 3), respectively. Both articles used curved mesial roots of mandibular molars for testing the glide path preparation times. Although Paleker et al. found no significant differences between ProGlide and G-files and D’Amario et al. demonstrated that G-files had lower mean values than Pathfiles, both concluded that glide path is achieved significantly faster with rotary glide path instruments than with K-files.

In comparing the glide path preparation time of different glide path files, two similar articles produced different results. In 2014, D’Agostino and Cantatore [87] compared glide path times of Pathfile and ProGlider when establishing the glide path on 100 resin blocks and 50 vestibular roots of maxillary first molars, and no significant differences were observed since the number of files in the Pathfile system was balanced by a slower advance with the ProGlider file. In 2015, Kirchhoff et al. [30] made the same comparison in mesial roots of mandibular molars but concluded that the glide path preparation time was significantly shorter for ProGlider (7.38 ± 1.73) than for Pathfile (20.61 ± 5.54). They explained the results by noting that ProGlider is only one instrument whereas the Profile system is composed of three instruments that are used to establish a glide path. The discrepancy in these findings could be attributed to the fact that D’Agostino also investigated the failure rate of the files and reused them multiple times, while Kirchhoff only used new files.

In 2018, Alfayate et al. [31] compared Pathfiles with the Profinder System in mesiobuccal root canals of mandibular molars with two types of angles of curvature (11–38° and 39–82°) and found out that Pathfiles were able to create a much faster glidepath (*p* = 0.004).

In 2021, Gambarini et al. [88] used upper first premolars with two canals and established a glide path in one canal with Edge GlidePath files and the other with ProGlider files. They concluded that Edge GlidePath files reached working length faster than ProGlider (9.25 ± 2.58 s vs. 14.87 ± 5.49 s).

Also in 2021, Han and Hou [89] showed that Hyflex GPF performs faster than Pathfile, but the results of this study should be deemed irrelevant in the matter. The article was a randomized clinical trial in which 80 patients had their molars with at least one curved root canal treated by one specialist. Patients were assigned in two groups based on the glide path files used—HyFlex EDM glide path files or PathFile—and the times were recorded. The problem is that in the Pathfile group, the time needed to change the files (PF1, 2 and 3) was also included.

Meanwhile, Alcalde et al. [90] tested two Brazilian files (X1 Glide path—reciprocating motion and Sequence Rotary File—continuous rotation) in sixty moderately curved canals of mandibular molars. Even though each file was used in three canals before being discarded, they concluded that rotary files reach working length faster than reciprocating glide path files due to the fact that reciprocating files “lose time” while disengaging the file.

Better results for reciprocating files were found by Vorster et al. in 2018 [91] when comparing the glide path preparation time of WaveOne Gold Glider (*n* = 15) with that of Pathfile (*n* = 15) and hand K-files (*n* = 15). The WaveOne Gold Glider group showed statistically significantly faster glide path preparation times compared to the PathFile and K-file groups, and the PathFile group in return showed statistically significantly faster preparation times compared with the K-file group. The subsequent shaping with WaveOne Gold Primary reached working length faster (*p* < 0.05) in all glide path groups (rotary or manual) than in the no glide path group.

In 2018, Zheng et al. [61] calculated the total working time of WaveOne Primary instrument with a glide path created by K-files, Pathfile or ProGlider in sixty mesial canals of mandibular first molars and concluded that the ProGlider + WaveOne Primary group was the fastest. However, the time recorded included the instrumentation phase, the cleaning of the instruments’ flutes, the working length confirmation and the irrigation protocol. The K-file group used #15 and #20 for glide path preparation, the Pathfile group used PF 1, 2 and 3, and the ProGlider group used the only ProGlider file, so it is unclear how much time was spent cleaning the flutes and irrigating between groups with multiple files and the Proglider group, which had only one file.

Similar results were achieved by Berutti et al. in 2014 [92] when they found out that the ProTaper Next X1 file reached working length faster when a glide path was created using the ProGlider rather than the Pathfile. The study used forty endo training blocks divided into two groups (*n* = 20 each) depending on the glide path preparation files used. The mean time required to complete shaping procedures with ProTaper Next X1 in the Profile group was 7.99 s compared with 5.91 s in the ProGlider group.

In 2018, Adıguzel and Tufenkci [93] compared shaping times of Reciproc and Reciproc Blue with a glide path created by C-pilot manual files and R-pilot reciprocating glide path files, or no prior glide path in 300 mesial canals of mandibular molars. They found that the shaping time was faster for the no glide path groups, and in the glide path groups, the manual glide path with C-pilot had a shorter time for completely shaping the canal. These results may appear contradictory to other findings, yet one potential explanation could be that the chronometer was never stopped if further instrumentation was deemed necessary after the three initial strokes, and it included the time required for cleaning the instrument, irrigation protocol, and patency confirmation. Two additional studies investigated this topic, employing similar comparisons and yielding analogous results. Ramyadharshini et al. [94] in 2020 and Jena et al. [95] in 2021 compared the shaping times of Twisted Files And Endostar E3 in adaptive motion and continuous rotation after a glide path was created up to a size 10, 15, or 20. Both articles used mandibular premolars for tests and concluded that the Twisted File system needed more time to reach working length and both systems needed more time to reach working length in smaller diameter glide paths, with the mean time for continuous rotation being less than that for adaptive motion.

Therefore, rotary glide path files (continuous or reciprocating) reduce the shaping time and glide path preparation time when compared to hand files.

### 3.4. Tortional Stress Resistance

Articles in this topic (*n* = 11) aimed to analyze the torsional strength of different glide path files or the influence of an established glide path on the torsional resistance of shaping files. The tests were performed using a torque meter with 3 mm of the instrument typically secured in place and a rotational force of 2 rpm applied, or with staging platforms to record peak torque while the instruments were working.

The first study was conducted on manual files by Kwak et al. [96] in 2014 when they compared the torsional strength of the C+ file, M Access file, Mani K-file, and NiTiFlex K-file. Each group (*n* = 10) was fixed at 3 mm, and clockwise rotations (2 rpm) were applied to the files in a straight state. The conclusion was that the C+ file had the highest torsional strength, but this may be because it had the largest cross-sectional area.

An interesting conclusion was drawn by Arias et al. [97] in 2015 when Profile and ProGlider were compared analyzing the peak torque while preparing eight canals of mesial roots of mandibular molars. It appears that multiple instrument systems generally demonstrate lower peak torque and peak force compared to single instrument systems. This could be attributed to single-instrument systems experiencing higher torque as a result of increased contact between the instruments’ larger flute diameter and dentine walls.

Another factor affecting the torsional strength of the instruments seems to be the pitch length, researched by Kwak et al. [67] and Al Raeesi et al. [98], who concluded that instruments with shorter pitch length have a higher torsional strength. Both studies used instruments fixed at 2, 4 and 6 mm and 2 mm, respectively, from the tip with a constant rotational speed of 2 rpm applied until instrument fracture occurred.

The type of motion also plays an important role in tortional strength, as Gavini et al. [99] found when comparing Scout RaCe and ProGlider in both continuous rotation and optimum glide path motion. A total of 48 files were divided into 2 groups (*n* = 24 each) and evaluated in both motions. All files were fixed at 3 mm from the tip, and the motion speed was 2 rpm in both types of motions. The ProGlider showed much greater torsional strength than ScoutRace regardless of the type of motion (*p* < 0.05), and the optimum glide path motion resulted in significantly lower torsional strength in both types of files compared to continuous rotation (*p* < 0.05).

Santos et al. [100] found higher tortional stress in R-pilot when compared to WaveOne Gold Glider. A number of 10 files each were used with 3 mm of their tip fixed and a counterclockwise rotation of 2 revolutions per minute applied. R-pilot instruments had a lower angle of rotation to fracture but a higher torque to failure than WaveOne Gold Glider.

Lopes et al. [101] had similar results when comparing R-pilot with WaveOne Gold Glider and ProGlider. A number of 10 instrument in each group were clamped at 3 mm from the tip, and the test was conducted in a counterclockwise rotation for R-pilot and WaveOne Gold Glider and in a clockwise rotation for Proglider, with a speed of 2 rpm maintained until instrument fracture. R-pilot had the highest torsional strength and the lowest angular deflection when compared to ProGlider and WaveOne Gold Glider (*p* = 0.008 and *p* = 0.0001, respectively). No differences in torsional strength and angular deflection were found between WaveOne Gold Glider and ProGlider (*p* > 0.05).

Yilmaz et al. [102] also compared R-pilot with Mtwo #10, ProGlider and WDV Rotate system. Twenty files from each group were clamped at 3 mm from the tip, and a rotation of 2 rpm was applied in a clockwise motion for Mtwo, Proglider and Rotate systems and counterclockwise for R-pilot. R-pilot showed the highest torsional strength of all groups, but the Rotate 15/04 glide path filed exhibited the highest angular distortion.

İnan and Keskin [103] analyzed the tortional strength of One-G when compared to ProGlider and Hyflex EDM. Fifteen files of each type were clamped at 3 mm from their tip and rotated in a clockwise direction at 2 rpm until fracture. No significant difference was found between Hyflex EDM and ProGlider in terms of torsional resistance values (*p* > 0.05), but while One G had the lowest torsional resistance (*p* < 0.05), Hyflex EDM showed the highest angle of rotation of all instruments (*p* < 0.05).

Regardless of the glide path technique used, Arias et al. [104] and Abu-Tahun et al. [105] showed that shaping instruments (ProTaper Gold and Hyflex, respectively) exhibited lower peak torque when an adequate glide path (higher than #15) was present. Protaper Gold files were used after a glide path was established with Pathfiles (*n* = 8) or ProGlider (*n* = 8) and Hyflex EDM files 25/08 were used after glide path preparation was performed with OneG in plastic blocks with a designated number of insertions (5, 10, 15, 20 or no glide path).

It is possible to conclude that instruments with a shorter pitch length have a higher torsional strength.

### 3.5. Apical Extrusion of Debris and/or Bacteria

Articles on this topic (*n* = 9) tried to analyze the apical extrusion of debris and/or bacteria resulting from different glide path files and/or subsequent use of shaping files with or without a glide path. When measuring extruded debris, a special apparatus is conceived to catch the debris, and the amount is weighted and compared. For measuring the extruded bacteria, the in vitro studies used root canals infected with *Enterococcus faecalis*, and the debris was collected in saline solutions, which was used to grow cultures.

Two articles researched the extrusion of bacteria. Dagna et al. [106] used sixty mandibular molars infected with a pure culture of *Entrerococcus faecalis*, mounted them in a collector apparatus, and established a glide path to compare the amount of extruded debris by K-files, Pathfile, G-file, ProGlider and One-G. The suspension resulting after establishing the glide path was collected and used to grow bacterial colonies. All glide path instruments tested were found to cause extrusion of bacteria, and they showed that K-files produced the most debris, which resulted in the highest count of bacterial colonies grown. Similar results were obtained by Low Pui-Yii et al. [107] with forty mandibular first molars when comparing manual glide path by K-files with rotary glide path by One-G and reciprocating glide path with WaveOne Gold Glider. The K-file group exhibited significantly more bacteria extrusion when compared to the rotary and reciprocating groups (*p* < 0.05). However, no significant differences were found between rotary and reciprocation instruments.

When comparing the extrusion of debris during the glide path preparation process, Ha et al. [108] came to the same conclusion. They used forty mandibular incisors mounted in a collector apparatus and measured the amount of debris extruded. The ProGlider group produced significantly less debris extrusion relative to the other groups (*p* < 0.05). No significant difference was found between OneG and ScoutRace groups, but overall debris extrusion was lower than that of the stainless-steel group (*p* < 0.05).

Similar results can be observed in articles where the total amount of debris from glidepath and shaping are compared. Zheng et al. [61] compared the amount of extruded debris from sixty mesial root canals of mandibular first molars with a glide path created by K-file, Pathfile and ProGlider and shaped with WaveOne, and found out that the K-file + WaveOne group produced the most debris. Also, Keskin et al. [109] collected debris from eighty mesial root canals of mandibular first molars with a glide path created by R-pilot, WaveOne Gold Glider, ProGlider, and K-file and shaped with Reciproc Blue, and found that the K-file + Reciproc Blue group produced the most debris (*p* < 0.05), while all groups caused debris extrusion. No significant differences were found between the amounts of debris caused by rotary files, regardless of the type of motion (*p* > 0.05).

Comprehensive research was conducted by Topçuoğlu et al. [110], who used WaveOne, Reciproc and One Shape to shape a root canal of ninety mandibular molars with no prior glidepath and one with a glide path established by Pathfile. All systems produced significantly more debris (*p* < 0.05) when shaping non-glide path root canals compared to those with a glide path. In the no glide path groups, One Shape produced smaller amounts of debris (*p* < 0.05), while in glide path groups, there were no significant differences between systems (*p* > 0.05).

When Gunes and Yesildal Yeter [19] researched apical extrusion after the glide path was established in sixty mandibular first molars with G-file, One-G, ProGlider, Pathfile and K-file, they found similar results, with K-file producing the most debris. However, when they shaped the glide path groups and the non-glide path group with WaveOne Gold Primary, they concluded that the glide path did not affect the apical extrusion of debris, as there were no statistically significant differences among groups. The same was true for Kırıcı et al. [111] when comparing extruded debris from thirty-six root canals of mandibular molars with no glide path or glide path created with WaveOne Gold Glider and ProGlider and shaped with WaveOne Gold Primary. The WaveOne Gold Glider + WaveOne Gold Primary group extruded less debris (*p* < 0.05), but there were no statistical differences between the ProGlider + WaveOne Gold Primary group and the group with no glide path created (*p* > 0.05).

One article can be suspected of bias as the comparisons were not standardized. Pawar et al. [112] compared the amount of extruded debris from root canals with a glide path created and then shaped with One Shape, WaveOne, and the SAF 1.5 mm file, and concluded that preparation with the SAF file produced the least amount of debris. The problem with the sample groups is that in the One Shape and WaveOne groups, the glide path was created with manual files up to a #20 K-file, and in the SAF group, the glide path was created with a rotary 20/04 file. All previous articles suggested that a manual glide path produces more debris than a rotary glide path, so the comparison would have been more precise if all groups received the same glide path, manual or rotary.

The results of these studies should be taken more as guidelines and not for certainty as in these in vitro experiments’ gravity and the absence of pressure from the periapical region might have an influence on the amount of extruded debris. Ultimately, glide path preparation and coronal preflaring seem to reduce the amounts of apical extruded debris and bacteria.

### 3.6. Defects in Dentine Walls

Articles on this topic (*n* = 8) aim to analyze if the glide path has any influence on shaping files producing cracks in dentine and the torque and screw-in forces of different glide path files. Excessive torque and increased screw-in forces can have adverse effects on dentine walls due to reactive forces within the dentin, potentially resulting in the formation of dentinal cracks.

Thu et al. [113] compared TrueNatomy Glider, ProGlider, Hyflex EDM and Dentcraft RE instruments (*n* = 14 each) in endo training blocks with one or two curvatures. TrueNatomy showed significantly higher torque than RE in canals with one curvature and ProGlider in canals with double curvatures (*p* < 0.05). EDM exhibited significantly higher screw-in force than TrueNatomy and RE in single-curved canals (*p* < 0.05).

Also, Kwak et al. [114] researched the torque and stress generated by shaping sixty mesio-buccal root canals of 3D printed teeth with One Curve after no glide path was established or a glide path was created with One-G, One Flare, or a combination of One-G and One Flare. Their findings suggested that establishing a glide path reduces stress and cumulative torque (*p* < 0.05). Consequently, they advise establishing a glide path to mitigate reactive forces in NiTi files and root canal dentin.

In another study, Kwak et al. [115] analyzed the total torque generated during shaping of sixty resin endo training blocks with WaveOne and WaveOne Gold after no prior glide path was established versus a glide path established with ProGlider. They demonstrated that the total torque produced by WaveOne Gold was significantly lowered when a glide path was present (*p* < 0.05), and the creation of a glide path did not produce significant changes in the maximum torque values for both file systems. WaveOne Gold where a glide path was present exhibited the lowest total torque generation among all groups (*p* < 0.05). WaveOne achieved a higher maximum torque compared to WaveOne Gold, regardless of the establishment of a glide path (*p* < 0.05).

Zanette et al. [46] studied the remaining dentin thickness in forty mesio-buccal roots after glide path established with Pathfile versus no glide path and shaping with ProTaper Universal up to F3 or F4 and found out that glide path is associated with greater remaining dentin thickness at 2 and 3 mm from the apex.

When studying dentin cracks, Topçuoğlu et al. [116] used Reciproc, WaveOne and ProTaper Next in 140 mandibular molars after a glide path created with Pathfile and no prior glide path. Analyses were carried out after shaping with #25 files and #40 files. No significant differences were found between instruments at #25 (*p* > 0.05), but Reciproc and WaveOne produced more apical cracks during shaping with #40 files (*p* < 0.05). However, when canals were prepared with #40 files, no propagation of existing cracks was caused (*p* > 0.05). They concluded that establishing a glide before canal preparation had no influence on the incidence of apical crack during preparation. Furthermore, increasing the apical preparation size may increase the incidence of apical crack during canal preparation.

On the other hand, Türker et al. [117] used ProTaper Next after ProGlider or no glide path in 45 mesial roots of mandibular first molars, Saber and Schäfer [118] used Reciproc after K-files or no glide path in 60 mesial roots of mandibular molars, Bürklein et al. [119] used ProTaper Next, F6 SkyTaper and One Shape after K-files or no glide path in 140 molars, and all of them concluded that the glide path had no impact on the incidence of dentinal cracks even in severely curved canals.

Finally, the glide path had no influence on the incidence of microcracks in dentine walls and had no real influence on the incidence of file separation or surface defects on shaping files.

### 3.7. File Separation

When instruments rotate inside a root canal, they are cyclically stressed, which can cause fatigue and lead to fracture. The main cause of instrument failure is the stress caused by bending and/or torsion. Torsional stress mostly appears when an instrument tip is larger than the canal section that it passes through. That is why articles in this topic (*n* = 5) aim to investigate if the creation of a glide path had any influence on file separation.

Berutti et al. [15] tested how many simulated root canals can be shaped with a ProTaper Universal S1 file with or without a glide path. The difference was significant (*p* < 0.001) and thus concluded that glide path has a major role in reducing the failure rate of rotary instruments. Similar results were obtained by Jonker et al. [120] when the research was focused on the number of simulated root canals a WaveOne file can shape after a glide path created by K-files, Pathfile or no glide path at all. It resulted that a greater number of simulated root canals could be shaped before the failure of WaveOne if a glide path was established with Pathfile (*p* < 0.01). The same was true for Ehrhardt et al. [121], who concluded that the glide path is responsible for a lower separation incidence of Mtwo instruments, after six endodontists performed a total of 556 treatments on maxillar and mandibular molars and bicuspids.

While researching alloy surface changes in shaping file after use in simulated curved canals with or without a glide path, Machado et al. [122] found out that WaveOne instruments tend to fracture, twist and crack more frequently when no glide path is present, and when a glide path is established, WaveOne and Reciproc instruments tend to crack less. On the contrary, Türker et al. [123] concluded that a prior glide path does not affect the surface topology of new and used WaveOne and One Shape single file systems. They used maxillary molars, and the files were divided into four subgroups where instruments were used in one or three root canals with or without an established glide path (*n* = 3).

The main point to be taken from this topic is that if clinicians create a rotary glide path or a manual pre-flaring in the root canal system that ensures there will not be a smaller diameter that the tip of the shaping file, they can avoid torsional stress and thus reduce the incidence of file separation.

### 3.8. Postoperative Pain Assessment

In this area of study, researchers conducted in vivo investigations (*n* = 5) to test the potential impact of glide path on postoperative pain reported by patients. Pain, being inherently subjective, presents challenges in standardization, measurement, and comparison across different individuals. Considering that shaping procedures are confined within the endodontic space, postoperative pain may be closely associated with the extrusion of debris and bacteria.

The overall conclusions drawn from these articles seem to be consistent with each other. Pasqualini et al. [124] concluded after a randomized clinical trial that a glide path with rotary files (Pathfile) causes less pain over the course of seven days than manual K-files. The postoperative pain prevalence curves in the Pathfile group showed a more favorable trend regarding time to pain resolution compared with the K-file group (*p* = 0.004). Keskin et al. [125] performed a similar research with a glide path created by hand (K-files), continuous rotation (ProGlider) and reciprocation (R-pilot) and monitored pain at 6, 12, 18, 24, 48 and 72 h. Conclusions were the same—establishing a glide path with rotary NiTi instruments (rotation or reciprocation) was associated with lower postoperative pain levels and incidence when compared to a manual glide path preparation, and no significant differences were observed between rotating and reciprocating instruments.

When Adıgüzel et al. [126] compared a continuous rotation glide path (One G) with reciprocating glide path (R-pilot) and no glide path at 24, 48 and 72 h intervals, differences were found at 24 h between the glide path with OneG files (less postoperative pain) and no glide path group, and no differences were found among all groups at 48 and 72 h.

Han and Hou [89] observed the pain levels over seven days after a glide path created with HyFlex EDM or Profile and shaping with ProTaper Next files. Both groups had higher levels of pain in the first day, which decreased over time, and the HyFlex group reported significantly less postoperative pain than Pathfile group patients overall (*p* < 0.001).

Only one study by Tufenkci et al. [127] studied intra-operatory pain, which could be more difficult to record especially after an inferior alveolar nerve block. The article compared R-pilot, WaveOne Gold Glider, One G and ProGlider files for the creation of a glide path and concluded that ProGlider caused the least intra-operatory pain, but the results should be taken with caution since the patients were anesthetized for the endodontic procedures.

Therefore, available evidence shows that the creation of a glide path tends to have a slight benefit on postoperative pain, especially immediate postoperative pain.

### 3.9. Scouting Ability and Performance

The scouting ability and overall performance of glide path rotary files have also been also measured in vitro. Articles in this topic (*n* = 4) measure parameters such as the preparation time, frequency of achieving working length, or cutting ability at different angles in gypsum.

DeDeus et al. [128] used moderately curved mandibular (*n* = 120) and maxillary (*n* = 120) extracted molars to test ScoutRaCe, ProDesign, Mtwo #10 and ProGlider files. ScoutRaCe performed more efficiently and reached working length in a higher percentage with less instrument separation than any other system. ProDesign had the lowest efficiency, and ProGlider had the highest rate of separation from all the systems tested.

In recent years, little research has been conducted in this particular area, and all data indicate the superiority of reciprocating motion. Pedullà et al. [129] used gypsum samples and measured the cutting efficiency of HyFlex EDM, One G, R-pilot, and WaveOne Gold Glider at 45°, 70° and 90° by determining the weight loss of the samples. R-pilot and WaveOne Gold Glider had a greater cutting efficiency than continuous rotation files, with R-pilot having the highest cutting ability at all angles.

Pereira et al. [130] concluded that WaveOne Gold Glider and R-pilot performed similarly regarding preparation time, frequency in reaching working length and plastic deformation rate when tested in sixty mesial roots of mandibular molars, and Campos et al. [131] concluded that both WaveOne Gold Glider and R-pilot are effective in reaching working length of mesial roots of mandibular molars. No instrument fractures were observed in the R-pilot group, while two fractures occurred in the WaveOne Gold Glider group (*p* > 0.05). Full working length was achieved in 29 canals (96.66%) and 28 canals (93.33%) with R-pilot and WaveOne Gold Glider instruments, respectively (*p* > 0.05).

Reciprocating glide path instruments have, in general, higher efficiency and cutting ability. Establishing a glide path may not have any influence on the ability of reciprocating files to reach working length.

## 4. Conclusions

Creating a glide path reduces root canal transportation, especially with rotary methods. Reciprocating and heat-treated files offer higher fatigue resistance and shorter preparation time. Instruments with shorter pitch lengths have greater torsional strength. Preparation and coronal preflaring reduce apical debris and bacteria. Glide paths do not affect dentine microcracks, file separation, or defects but reduce immediate postoperative pain and improve cutting ability. Randomized trials are needed to assess their impact on treatment outcomes.

## Figures and Tables

**Table 1:** Comparison of the centering ability and root canal transportation of two or more glide rotary Ni-Ti files.

Author/Year	Specimen	Comparison Groups	Methods for Evaluation	Conclusions
de Oliveira Alves et al., 2012 [17]	Mesial canals of mandibular molars (*n* = 45)	Group 1—K-file Group 2—first 3 files of MTwo system Group 3—PathFile	Superimposed X-rays	No apical transportation observed in any group
Pasqualini et al., 2012 [26]	Buccal root canals of upper first molars (*n* = 16)	Group 1—Pathfile Group 2—K-file	Cone beam geometry system	Pathfile does a better job in preserving the original canal anatomy and tend to cause less canal aberrations
Natasha C.C. Ajuz et al., 2013 [27]	S-shaped training blocks (*n* = 60)	Group 1—K-file Group 2—Pathfile Group 3—Scout RaCe	Superimposed stereomicroscopic images	- Rotary Ni-Ti instruments produced less deviation - Scout RaCe caused significantly less modification than Pathfiles
D’Amario et al., 2013 [28]	Mesial canals of mandibular molars (*n* = 45)	Group 1—G-file Group 2—Pathfile Group 3—K-file	Digital double X-ray technique	No significant differences found between groups
Anil Dhingra and Nayasha Manchanda, 2014 [29]	Curved mesial roots of mandibular first molars (*n* = 100)	Group 1—Pathfile Group 2—VGP2	CBCT	Pathfiles caused significantly less transportation at all levels
Kirchhoff et al., 2015 [30]	Curved mesial canals of mandibular molars (*n* = 40)	Group 1—ProGlider Group 2—Pathfile	Micro CT-scan	Similar apical transportation observed in both groups
Paleker and van der Vyver, 2016 [24]	Separate mesiobuccal and mesiolingual canals with 25–30° curvature (*n* = 90)	Group 1—K-file Group 2—G-file 1 and 2 Group 3—ProGlider	Micro CT	- K-file preparation was less centered at all levels - ProGlider showed superior overall centering ability
Alfayate et al., 2018 [31]	Mesiobuccal root canals of mandibular molars with curvature between 11–82° (*n* = 60)	Group 1a—11–38° Pathfile Group 1b—11–38° ProFinder Group 2a—39–82° Pathfile Group 2b—39–82° ProFinder	Digital X-rays	Both systems were effective in maintaining original root canal anatomy
Shalan and Al-Huwaizi, 2018 [32]	Resin blocks with L-shaped canal (*n* = 40)	Group 1—WOGG Group 2—ProGlider Group 3—Pathfile Group 4—K-file	Superimposed digital photographic images	- WaveOne Gold Glider showed less transportation in apical third - No significant differences between ProGlider, Pathfile and K-file
van der Vyver et al., 2018 [33]	Mesiobuccal root canals of maxillary molars (*n* = 135)	Group 1—K-file Group 2—One-G Group 3—ProGlider	Micro-CT scans	One-G and ProGlider showed less canal transportation on all levels examined
Nazari Moghadam et al., 2018 [34]	Maxillary molars with separate MB2 (*n* = 66)	Group 1—ProGlider Group 2—Scout Race Group 3—M3 pro Glide path	CBCT scan	ProGlider showed less transportation in apical third
Htun et al., 2019 [35]	Mandibular incisors (*n* = 30)	Group 1—HyFlex EDM in OGM Group 2—Hyflex EDM in CR Group 3—K-files	Micro-CT scans	Canal transportation at 1 and 3 mm from the apex was insignificant between groups
Aydın et al., 2019 [36]	Mandibular first molars with 2 separate mesial canals (*n* = 24)	Group 1—ProGlider Group 2—WOGG Group 3—R-Pilot	Micro-CT analysis	R-pilot and WOGG had less transportation in coronal and middle third
G. Česaitienė et al., 2019 [37]	Mesio and distobuccal canals of maxillary molars and mesiobuccal and lingual canals of mandibular molars (*n* = 36)	Group 1—Pathfile 1 and 2 Group 2—Pathfile 2 Group 3—ProGlider	Micro-CT scans	All three groups performed very similar without significant differences
Htun et al., 2020 [38]	Double-curved resin canals (*n* = 60)	Group 1—Hyflex EDM #10/05 in CR Group 2—Hyflex EDM #10/05 in OGM Group 3—Hyflex EDM #15/03 in CR Group 4—Hyflex EDM #15/03 in OGM Group 5—MANI Glidepath file #13/04 in CR Group 6—MANI Glidepath file #13/04 in OGM	Micro-CT scan	CR and OGM generated similar transportation in both files used
Aflaki S et al., 2020 [39]	Mandibular first and second molars	Group 1—K-file Group 2—Pathfile Group 3—Scout RaCe	CBCT	- K-file caused significantly higher canal transportation - No significant differences between Pathfile and Scout Race
Liu et al., 2021 [40]	Mesial root canals of mandibular molars (*n* = 30)	Group 1—K-files Group 2—MANI Mechanical Glide Path files in OGM	Micro-CT scan	Group 2 showed significantly lower canal transportation
M. Aminsobhani et al., 2022 [41]	S-Shaped canal simulator blocks (*n* = 100)	Group 1—Scout RaCe Group 2—One-G Group 3—Pathfile Group 4—Neolix GPS Group 5—K-file	Superimposed photos in Photoshop	- K-file showed significantly more transportation - No difference between rotary groups
Yeniçeri Özata et al., 2023 [42]	Mandibular molars with separate mesial canals	Group 1—TRN-G Group 2—WOGG Group 3—ProGlider	Micro-CT Scan	TRN-G showed significantly greater transportation than the other instruments

**Abbreviations:** VGP, 2 V Taper Glide Path File; CBCT, Cone Beam Computed Tomography; Micro-CT, micro–computed tomographic; WOGG, WaveOne Glod Glider; OGM, Optimum Glidepath Motion; CR, Continuous Rotation; TRN-G, TrueNatomy Glider.

**Table 2:** Analyzing the centering ability and root canal transportation of shaping files with or without a glide path.

Author/Year	Specimen	Comparison Groups	Methods for Evaluation	Conclusions
Uroz-Torres et al., 2009 [43]	Mesiobuccal canals of mandibular molars (*n* = 40)	Group 1—MC-K-file + Mtwo Group 2—MC-Mtwo Group 3—SC-K-file + Mtwo Group 4—SC-Mtwo	Superimposed X-rays	Creating a manual glidepath did not influence apical transportation of Mtwo instruments
Berutti et al., 2012 [44]	Endo training blocks (*n* = 30)	Group 1—Pathfile + WaveOne Group 2—WaveOne	Superimposed digital images	Canal transportation significantly reduced with glide path
Nazarimoghadam et al., 2014 [45]	Resin blocks with 60° curvature (*n* = 30)	Group 1—K-file + Reciproc Group 2—Reciproc	Superimposed digital images	Glide path reduced transportation in apical third
Zanette et al., 2014 [46]	Mesiobuccal roots of maxillary molars (*n* = 40)	Group 1—Pathfiles + PTU Group 2—PTU	Superimposed X-rays	Glidepath did not influence apical transportation
Elnaghy and Elsaka, 2014 [47]	Mesiobuccal canals of mandibular first molars (*n* = 60)	Group 1—ProGlider + PTN Group 2—Profile + PTN Group 3—PTN	CBCT	The first group showed significantly lower transportation
Dhingra et al., 2015 [48]	Mandibular first molars (*n* = 100)	Group 1—WaveOne Group 2—Pathfile + WaveOne	CBCT	Group 2 showed significantly reduced canal transportation
Yilmaz et al., 2017 [49]	S-shaped endo training blocks (*n* = 40)	Group 1—Pathfile + WaveOne Group 2—WaveOne	Superimposed digital images	Glide path has been shown to improve the centering ability of WaveOne and reduced the incidents of canal aberrations
Keskin et al., 2018 [50]	S-shaped blocks (*n* = 30)	Group 1—ProGlider + Reciproc Blue Group 2—Reciproc Blue	Superimposed digital images	Group 2 showed significantly greater transportation

**Abbreviations:** MC, moderate curvature; SC, severe curvature, PTU, ProTaper Universal, PTN, ProTaper Next, CBCT, Cone Beam Computed Tomography.

**Table 3:** Comparison of the centering ability and root canal transportation of different shaping files after glide path.

Author/Year	Specimen	Comparison Groups	Methods for Evaluation	Conclusions
Bȕrklein et al., 2014 [51]	S-shaped canals in resin blocks (*n* = 120)	Group 1—Reciproc R25 Group 2—Reciproc R25+ PF Group 3—WaveOne 25 Group 4—WaveOne 25 + PF Group 5—Hyflex CM Group 6—Hyflex CM + PF Group 7—F360 Group 8—F360 + PF Group 9—OneShape Group 10—OneShape + PF	Superimposed digital images	- Glide path did not influence the centering ability of the systems used - Instruments with smaller taper maintained the original canal curvature better than the ones with greater taper
Coelho et al., 2016 [18]	Mandibular molars with separate canals in mesial root (*n* = 60)	Group 1—WaveOne Group 2—WaveOne + K-file Group 3—Reciproc Group 4—Reciproc + K-file	Superimposed X-rays	Glide path had no influence in the centering ability of those systems
Hage et al., 2020 [52]	Maxillary and mandibular premolars (*n* = 120)	Group 1—R25 Group 2—R25 + PF Group 3—R25 Blue Group 4—R25 Blue + PF	CBCT	When PF was used, less transportation and better centering occurred in both groups
Biasillo et al., 2021 [53]	S-shaped canals in resin blocks (*n* = 40)	Group 1—OneCurve + OneG Group 2—OneCurve Group 3—R25 Blue Group 4—R25 Blue + R-pilot	Superimposed digital images	- Glide path improved the centering ability in the apical third - R25 Blue performed better
Alqahtani and AbuMostafa, 2021 [54]	Mesiobuccal canals of mandibular molars (*n* = 48)	Group 1—Race Evo NGP, NCF Group 2—Race Evo GP, NCF Group 3—Race Evo GP, CF Group 4—EdgeSeq NGP, NCF Group 5—EdgeSeq GP, NCF Group 6—EdgeSeq GP, CF	Micro-CT	There were no significant differences regarding transportation and centering ability among the groups tested
Seda Falakaloğlu et al., 2022 [55]	Resin J-shaped root canals (*n* = 34)	Group 1—TEM tg + TEM M25 Group 2—WOGG + WOG Primary	Superimposed digital images	Both systems showed respect for the original canal curvature
Alovisi et al., 2022 [56]	Mesiobuccal canals of maxillary first molars (*n* = 30)	Group 1—PG + PTN X1, X2 Group 2—WOGG +WOG	Micro-CT	Both systems produce well-centered preparations
L. Shi, Y. Yang, J. Wan et al., 2022 [57]	J-shaped endo training blocks (*n* = 80)	Group 1—OneCurve + NGP Group 2—OneCurve + PF Group 3—OneCurve + PG Group 4—OneCurve + WOGG Group 5—R25Blue + NGP Group 6—R25Blue + PF Group 7—R25Blue + PG Group 8—R25Blue + WOGG	Superimposed digital images	- PG and WOGG subgroups produced less transportation in both major groups - One Curve + PG and WOGG, produced the least transportation

**Abbreviations:** PF, Pathfile; CBCT, Cone Beam Computed Tomography; R25, Reciproc #25, NGP; No Glide Path; NCF, No Coronal Preflaring; GP, Glide Path; CF, Coronal Preflaring; CT, computer tomography; TEM tg, T Endo Must true glidepath file; TEM M25, T Endo Must 25/06 file; WOGG, WaveOne Gold Glider; WOG—WaveOne Gold; PG, Proglider; PTN, ProTaper Next.

**Table 4:** Analyzing entering ability and root canal transportation of a shaping file after different glide path files.

Author/Year	Specimen	Comparison Groups	Methods for Evaluation	Conclusions
de Carvalho et al., 2015 [58]	Mesial root canals of mandibular molars (*n* = 52)	Group 1—R25 + K-file Group 2—R25 + NGP Group 3—R25 + PF Group 4—no preparation	CBCT	All glide path techniques exhibited minimal apical transportation
Shi and Wagle, 2017 [59]	J-shaped endo training blocks (*n* = 60)	Group 1—G-file + Hyflex CM Group 2—PF + Hyflex CM Group 3—Hyflex GPF + Hyflex CM	Superimposed digital images	Shaping with Hyflex after glide path preparation produced no significant difference
Vorster et al., 2018 [60]	Mesiobuccal canals of mandibular molars (*n* = 60)	Group 1—K-file + PWOG Group 2—PF + PWOG Group 3—WOGG + PWOG Group 4—NGP + PWOG	Micro-CT	PWOG centering ability and transportation was not influenced by GP/NGP
Zheng et al., 2018 [61]	Mesial canals of mandibular first molars (*n* = 60)	Group 1—K-file + WaveOne Group 2—PF + WaveOne Group 3—PG + WaveOne	Micro-CT	PG + WaveOne showed the least canal transportation
Alovisi et al., 2017 [62]	Maxillary first molars (*n* = 45)	Group 1—PF + PTN X1, X2 Group 2—PG + PTN X1, X2 Group 3—K-file + PTN X1, X2	Micro-CT	PG + PTN group had minimum transportation values

**Abbreviations:** R25, Reciproc file #25; NGP, No Glide Path; PF, PathFile; CBCT, Cone Beam Computed Tomography; PWOG, WaveOne Gold Primary file; WOGG, WaveOne Gold Glider; CT, computer tomography; GP, GlidePath; PG, Proglider; PTN, ProTaper Next.

**Table 5:** Comparison of cyclic fatigue resistance of different glide path files.

Author/Year	Files Compared	Method for Determination	Criteria Researched	Results
Gambarini et al., 2013 [68]	K-file in M4 handpiece/Pathfile	SS canal with 60° curvature and 5 mm radius	TTF	K-file > Pathfile
Sung et al., 2014 [69]	G-file #1,2/PF #1,2,3	SS canal with 90° curvature and 3 mm radius	NCF	PF1 > PF2 > G1 > PF3 > G2
Capar et al., 2015 [70]	PF/G-file/Scout RaCe/Hyflex GPF/PG	SS canal with 90° curvature and 3 mm radius	NCF	Hyflex > G-files > PG > PF > Scout RaCe
Uslu et al., 2016 [71]	PG/One G	SS canal with 60° curvature and 5 mm radius	TTF, NCF	TTF PG > One G NCF PG > One G
Özyürek et al., 2016 [72]	Used and new PF/PG	SS canal with 60° curvature and 5 mm radius	NCF	PF > PG New > used (*p* > 0.05)
Uslu et al., 2017 [73]	R-pilot/Hyflex EDM/PF	SS double-curved canal	NCF	R-pilot > Hyflex > PF
Yilmaz et al., 2017 [74]	Hyflex EDM/One G/PG	SS canal with 60° curvature and 5 mm radius, 1 and 2 curves	NCF	1 curve > 2 curves Hyflex > PG > One G in both curves
Özyürek et al., 2018 [75]	R-pilot/WOGG	SS canal with 60° curvature and 5 mm radius	TTF	R-pilot > WOGG
Topçuoğlu et al., 2018 [76]	R-pilot/WOGG	SS canal with 45° and 60° curvature and 5 mm radius	TTF	45° no difference 60° WOGG > R-pilot
Serefoglu et al., 2018 [77]	WOGG/R-pilot/PG	SS canal with 90° curvature and 3 mm radius	NCF	WOGG > R-pilot > PG
Keskin et al., 2018 [78]	R-pilot/WOGG/PG	SS canal with 60° curvature and 5 mm radius	TTF	WOGG > R-pilot > PG
Yilmaz et al., 2018 [79]	One G/PG/Hyflex EDM/R-pilot	SS canal with 60° curvature and 5 mm radius	TTF	R-pilot > Hyflex > PG > One G
Nishijo et al., 2018 [80]	Hyflex EDM/Hyflex GPF	SS canal with 60° curvature and 5 mm radius	TTF	Hyflex EDM > Hyflex GPF
Topçuoğlu et al., 2018 [81]	PF/Scout RaCe/PG	SS double-curved canal	NCF	PG > PF and Scout RaCe
Kırıcı and Kuştarcı, 2019 [82]	WOGG/PG/One G	SS double-curved canal	NCF	WOGG > PG > One G
Lee et al., 2019 [83]	PG/One G/Edge glidepath	SS canal with 90° curvature and 3 mm radius	TTF	Edge > PG > One G
S Oh et al., 2022 [84]	TRN glider/V taper 2H/Hyflex EDM	SS canal with 60° curvature and 1.5 mm radius	NCF	V taper > TRN and Hyflex
JNR Martins et al., 2022 [85]	PG/Edge glidepath/R-pilot	SS canal with 86° curvature and 6 mm radius	TTF	R-pilot > PG > Edge

**Abbreviations:** SS, Stainless Steel; TTF, Time to Fracture; PF, PathFile; NCF, Number of Cycles to Fracture; PG, ProGlider; WOGG, WaveOne Gold Glider; TRN, TrueNatomy.

## Data Availability

No new data were created or analyzed in this study. Data sharing is not applicable to this article.

## References

[1] Schilder H. (1974). Cleaning and Shaping the Root Canal. Dent. Clin. North Am..

[2] PETERS O. (2004). Current Challenges and Concepts in the Preparation of Root Canal Systems: A Review. J. Endod..

[3] Mullaney T.P. (1979). Instrumentation of Finely Curved Canals. Dent. Clin. N. Am..

[4] Goerig A.C., Michelich R.J., Schultz H.H. (1982). Instrumentation of Root Canals in Molar Using the Step-down Technique. J. Endod..

[5] Roane J., Sabala C., Duncansonjr M. (1985). The “Balanced Force” Concept for Instrumentation of Curved Canals. J. Endod..

[6] Goldberg F., Araujo J.A. (1997). Comparison of Three Instruments in the Preparation of Curved Root Canals. Dent. Traumatol..

[7] Walia H., Brantley W.A., Gerstein H. (1988). An Initial Investigation of the Bending and Torsional Properties of Nitinol Root Canal Files. J. Endod..

[8] Schäfer E., Chong B.S. (2017). Preparation of the Root Canal System. Harty’s Endodontics in Clinical Practice.

[9] Berutti E., Cantatore G., Castellucci A., Chiandussi G., Pera F., Migliaretti G., Pasqualini D. (2009). Use of Nickel-Titanium Rotary PathFile to Create the Glide Path: Comparison with Manual Preflaring in Simulated Root Canals. J. Endod..

[10] Hulsmann M., Peters O.A., Dummer P.M.H. (2005). Mechanical Preparation of Root Canals: Shaping Goals, Techniques and Means. Endod. Top..

[11] Spanaki-Voreadi A.P., Kerezoudis N.P., Zinelis S. (2006). Failure Mechanism of ProTaper Ni–Ti Rotary Instruments during Clinical Use: Fractographic Analysis. Int. Endod. J..

[12] Martín B., Zelada G., Varela P., Bahillo J.G., Magán F., Ahn S., Rodríguez C. (2003). Factors Influencing the Fracture of Nickel-Titanium Rotary Instruments. Int. Endod. J..

[13] Peters O.A., Peters C.I., Schönenberger K., Barbakow F. (2003). ProTaper Rotary Root Canal Preparation: Assessment of Torque and Force in Relation to Canal Anatomy. Int. Endod. J..

[14] Kobayashi C., Yoshioka T., Suda H. (1997). A New Engine-Driven Canal Preparation System with Electronic Canal Measuring Capability. J. Endod..

[15] Berutti E., Negro A., Lendini M., Pasqualini D. (2004). Influence of Manual Preflaring and Torque on the Failure Rate of ProTaper Rotary Instruments. J. Endod..

[16] West J. (2006). Endodontic Update 2006. J. Esthet. Restor. Dent..

[17] de Oliveira Alves V., da Silveira Bueno C.E., Cunha R.S., Pinheiro S.L., Fontana C.E., de Martin A.S. (2012). Comparison among Manual Instruments and PathFile and Mtwo Rotary Instruments to Create a Glide Path in the Root Canal Preparation of Curved Canals. J. Endod..

[18] Coelho M.S., Fontana C.E., Kato A.S., de Martin A.S., da Silveira Bueno C.E. (2016). Effects of Glide Path on the Centering Ability and Preparation Time of Two Reciprocating Instruments. Iran. Endod. J..

[19] Gunes B., Yesildal Yeter K. (2018). Effects of Different Glide Path Files on Apical Debris Extrusion in Curved Root Canals. J. Endod..

[20] Bürklein S., Schäfer E. (2013). Critical Evaluation of Root Canal Transportation by Instrumentation. Endod. Top..

[21] West J.D. (2010). The Endodontic Glidepath: “Secret to Rotary Safety”. Dent. Today.

[22] Van der Vyver P. (2010). Creating a Glide Path for Rotary NiTi Instruments: Part One. Int. Dent. SA.

[23] Bergmans L., Van Cleynenbreugel J., Wevers M., Lambrechts P. (2001). Mechanical Root Canal Preparation with NiTi Rotary Instruments: Rationale, Performance and Safety. Status Report for the American Journal of Dentistry. Am. J. Dent..

[24] Paleker F., van der Vyver P.J. (2016). Comparison of Canal Transportation and Centering Ability of K-Files, ProGlider File, and G-Files: A Micro-Computed Tomography Study of Curved Root Canals. J. Endod..

[25] Page M.J., McKenzie J.E., Bossuyt P.M., Boutron I., Hoffmann T.C., Mulrow C.D., Shamseer L., Tetzlaff J.M., Akl E.A., Brennan S.E. (2021). The PRISMA 2020 Statement: An Updated Guideline for Reporting Systematic Reviews. BMJ.

[26] Pasqualini D., Bianchi C.C., Paolino D.S., Mancini L., Cemenasco A., Cantatore G., Castellucci A., Berutti E. (2012). Computed Micro-Tomographic Evaluation of Glide Path with Nickel-Titanium Rotary PathFile in Maxillary First Molars Curved Canals. J. Endod..

[27] Ajuz N.C.C., Armada L., Gonçalves L.S., Debelian G., Siqueira J.F. (2013). Glide Path Preparation in S-Shaped Canals with Rotary Pathfinding Nickel-Titanium Instruments. J. Endod..

[28] D’Amario M., Baldi M., Petricca R., De Angelis F., El Abed R., D’Arcangelo C. (2013). Evaluation of a New Nickel-Titanium System to Create the Glide Path in Root Canal Preparation of Curved Canals. J. Endod..

[29] Dhingra A. (2014). Modifications in Canal Anatomy of Curved Canals of Mandibular First Molars by Two Glide Path Instruments Using C BCT. J. Clin. Diagnostic Res..

[30] Kirchhoff A.L., Chu R., Mello I., Garzon A.D.P., dos Santos M., Cunha R.S. (2015). Glide Path Management with Single- and Multiple-Instrument Rotary Systems in Curved Canals: A Micro-Computed Tomographic Study. J. Endod..

[31] Alfayate R.P., Mercade M., Rojas J.V., Luaña R.E., Pereda A.A., Algar J., Cabello R.C. (2018). Comparison of PathFile and ProFinder Systems to Create a Glide Path in Curved Root Canals. Eur. Endod. J..

[32] Shalan L.A., Al-Huwaizi H.F. (2018). Evaluation of Canal Transportation after Using Different Types Rotary Glide Path Files. Iran. Endod. J..

[33] van der Vyver P.J., Paleker F., Vorster M., de Wet F.A. (2019). Micro-Computed Tomographic Evaluation of Two Single Rotary Glide Path Systems. Int. Endod. J..

[34] Nazari Moghadam K., Farajian Zadeh N., Labbaf H., Kavosi A., Farajian Zadeh H. (2019). Negotiation, Centering Ability and Transportation of Three Glide Path Files in Second Mesiobuccal Canals of Maxillary Molars: A CBCT Assessment. Iran. Endod. J..

[35] Htun P.H., Ebihara A., Maki K., Kimura S., Nishijo M., Tokita D., Okiji T. (2020). Comparison of Torque, Force Generation and Canal Shaping Ability between Manual and Nickel-Titanium Glide Path Instruments in Rotary and Optimum Glide Path Motion. Odontology.

[36] Aydın Z.U., Keskin N.B., Özyürek T., Geneci F., Ocak M., Çelik H.H. (2019). Microcomputed Assessment of Transportation, Centering Ratio, Canal Area, and Volume Increase after Single-File Rotary and Reciprocating Glide Path Instrumentation in Curved Root Canals: A Laboratory Study. J. Endod..

[37] Česaitienė G., Venskutonis T., Mačiulskienė V., Cicėnas V., Samaitis V., Jasiūnienė E. (2019). Micro-Computed Tomography (Micro-CT) Evaluation of Effects of Different Rotary Glide Path Techniques on Canal Transportation and Centering in Curved Root Canals. Med. Sci. Monit..

[38] Htun P.H., Ebihara A., Maki K., Kimura S., Nishijo M., Kyaw M.S., Okiji T. (2021). Comparison of Torque, Screw-in Force, and Shaping Ability of Glide Path Instruments in Continuous Rotation and Optimum Glide Path Motion. J. Endod..

[39] Aflaki S., Boyerahmadi E., Talaei A., Safari M.R., Mohammadpour M., Mohammadi N., Adel M. (2020). In Vitro Transportation of Curved Canals Following Glide Path Preparation by Path File and Scout RaCe Rotary Systems versus Manual Instrumentation Using Cone-Beam Computed Tomography. Front. Dent..

[40] Liu J.-Y., Zhou Z.-X., Tseng W.-J., Karabucak B. (2021). Comparison of Canal Transportation and Centering Ability of Manual K-Files and Reciprocating Files in Glide Path Preparation: A Micro-Computed Tomography Study of Constricted Canals. BMC Oral Health.

[41] Aminsobhani M., Hamidzadeh F., Rezaei Avval A., Merrikhi F., Sadri E. (2022). Evaluation of the Canal Transportation Following Glide Path Preparation with Different Rotary Systems: A Comparative Study. Sci. World J..

[42] Yeniçeri Özata M., Falakaloğlu S., Keleş A., Adıgüzel Ö., Gündoğar M. (2023). Evaluation of Shaping Ability of Different Glide Path Instruments: A Micro-Computed Tomography Study. BMC Oral Health.

[43] Uroz-Torres D., González-Rodríguez M.P., Ferrer-Luque C.M. (2009). Effectiveness of a Manual Glide Path on the Preparation of Curved Root Canals by Using Mtwo Rotary Instruments. J. Endod..

[44] Berutti E., Paolino D.S., Chiandussi G., Alovisi M., Cantatore G., Castellucci A., Pasqualini D. (2012). Root Canal Anatomy Preservation of WaveOne Reciprocating Files with or without Glide Path. J. Endod..

[45] Nazarimoghadam K., Daryaeian M., Ramazani N. (2014). An in Vitro Comparison of Root Canal Transportation by Reciproc File with and without Glide Path. J. Dent..

[46] Zanette F., Grazziotin-Soares R., Flores M.E., Camargo Fontanella V.R., Gavini G., Barletta F.B. (2014). Apical Root Canal Transportation and Remaining Dentin Thickness Associated with ProTaper Universal with and without PathFile. J. Endod..

[47] Elnaghy A.M., Elsaka S.E. (2014). Evaluation of Root Canal Transportation, Centering Ratio, and Remaining Dentin Thickness Associated with ProTaper Next Instruments with and without Glide Path. J. Endod..

[48] Dhingra A., Nagar N., Sapra V. (2015). Influence of the Glide Path on Various Parameters of Root Canal Prepared with WaveOne Reciprocating File Using Cone Beam Computed Tomography. Dent. Res. J..

[49] Yilmaz A., Kucukay E.S., Istektepe M., Sisli S.N., Ersev H., Karagoz-Kucukay I. (2017). Comparison of the Shaping Ability of WaveOne Reciprocating Files with or without Glide Path in Simulated Curved S-Shaped Root Canals. J. Int. Soc. Prev. Community Dent..

[50] Keskin C., Sarıyılmaz E., Demiral M. (2018). Shaping Ability of Reciproc Blue Reciprocating Instruments with or without Glide Path in Simulated S-Shaped Root Canals. J. Dent. Res. Dent. Clin. Dent. Prospect..

[51] Bürklein S., Poschmann T., Schäfer E. (2014). Shaping Ability of Different Nickel-Titanium Systems in Simulated S-Shaped Canals with and without Glide Path. J. Endod..

[52] Hage W., Zogheib C., Bukiet F., Sfeir G., Khalil I., Gergi R., Naaman A. (2020). Canal Transportation and Centring Ability of Reciproc and Reciproc Blue with or without Use of Glide Path Instruments: A CBCT Study. Eur. Endod. J..

[53] Biasillo V., Castagnola R., Colangeli M., Panzetta C., Minciacchi I., Plotino G., Staffoli S., Marigo L., Grande N.M. (2022). Comparison of Shaping Ability of the Reciproc Blue and One Curve with or without Glide Path in Simulated S-Shaped Root Canals. Restor. Dent. Endod..

[54] Alqahtani O., AbuMostafa A. (2021). Effect of Glide Path and Coronal Flaring on the Centering Ability and Transportation of Root Canals: Micro-CT In Vitro Study. J. Contemp. Dent. Pract..

[55] Falakaloğlu S., Iriboz E. (2022). Comparison of Shaping Ability of T-Endo MUST and WaveOne Gold with Glide Path Instruments: An In Vitro Study. Turk. Klin. J. Dent. Sci..

[56] Alovisi M., Pasqualini D., Scotti N., Carpegna G., Comba A., Bernardi M., Tutino F., Dioguardi M., Berutti E. (2022). Micro-CT Evaluation of Rotary and Reciprocating Glide Path and Shaping Systems Outcomes in Maxillary Molar Curved Canals. Odontology.

[57] Shi L., Yang Y., Wan J., Xie W., Yang R., Yao Y. (2022). Shaping Ability of Rotary and Reciprocating Single-File Systems in Combination with and without Different Glide Path Techniques in Simulated Curved Canals. J. Dent. Sci..

[58] de Carvalho G.M., Sponchiado Junior E.C., Garrido A.D.B., Lia R.C.C., Garcia L.D.F.R., Marques A.A.F. (2015). Apical Transportation, Centering Ability, and Cleaning Effectiveness of Reciprocating Single-File System Associated with Different Glide Path Techniques. J. Endod..

[59] Shi L., Wagle S. (2017). Comparing the Centering Ability of Different Pathfinding Systems and Their Effect on Final Instrumentation by Hyflex CM. J. Endod..

[60] Vorster M., van der Vyver P.J., Paleker F. (2018). Canal Transportation and Centering Ability of WaveOne Gold in Combination with and without Different Glide Path Techniques. J. Endod..

[61] Zheng L., Ji X., Li C., Zuo L., Wei X. (2018). Comparison of Glide Paths Created with K-Files, PathFiles, and the ProGlider File, and Their Effects on Subsequent WaveOne Preparation in Curved Canals. BMC Oral Health.

[62] Alovisi M., Cemenasco A., Mancini L., Paolino D., Scotti N., Bianchi C.C., Pasqualini D. (2017). Micro-CT Evaluation of Several Glide Path Techniques and ProTaper Next Shaping Outcomes in Maxillary First Molar Curved Canals. Int. Endod. J..

[63] Özyürek T., Uslu G., Yılmaz K., Gündoğar M. (2018). Effect of Glide Path Creating on Cyclic Fatigue Resistance of Reciproc and Reciproc Blue Nickel-Titanium Files: A Laboratory Study. J. Endod..

[64] Uslu G., İnan U. (2019). Effect of Glide Path Preparation with PathFile and ProGlider on the Cyclic Fatigue Resistance of WaveOne Nickel-Titanium Files. Restor. Dent. Endod..

[65] Ates A.A., Arican B., Ounsi H.F. (2020). Influence of Rotational Speed and Glide Path on Cyclic Fatigue Resistance of XP-Endo Shaper. Niger. J. Clin. Pract..

[66] Scherer A.S., Bier C.A.S., Vanni J.R. (2023). Effect of Glide Path Instruments in Cyclic Fatigue Resistance of Reciprocating Instruments after Three Uses. Braz. Dent. J..

[67] Kwak S.W., Ha J.-H., Lee C.-J., El Abed R., Abu-Tahun I.H., Kim H.-C. (2016). Effects of Pitch Length and Heat Treatment on the Mechanical Properties of the Glide Path Preparation Instruments. J. Endod..

[68] Gambarini G., Plotino G., Sannino G., Grande N.M., Giansiracusa A., Piasecki L., da Silva Neto U.X., Al-Sudani D., Testarelli L. (2015). Cyclic Fatigue of Instruments for Endodontic Glide Path. Odontology.

[69] Sung S.Y., Ha J.-H., Kwak S.-W., Abed R.E., Byeon K., Kim H.-C. (2014). Torsional and Cyclic Fatigue Resistances of Glide Path Preparation Instruments: G-File and PathFile. Scanning.

[70] Capar I.D., Kaval M.E., Ertas H., Sen B.H. (2015). Comparison of the Cyclic Fatigue Resistance of 5 Different Rotary Pathfinding Instruments Made of Conventional Nickel-Titanium Wire, M-Wire, and Controlled Memory Wire. J. Endod..

[71] Uslu G., Özyürek T., İnan U. (2016). Comparison of Cyclic Fatigue Resistance of ProGlider and One G Glide Path Files. J. Endod..

[72] Özyürek T., Uslu G., İnan U. (2017). A Comparison of the Cyclic Fatigue Resistance of Used and New Glide Path Files. J. Endod..

[73] Uslu G., Özyürek T., Yılmaz K., Gündoğar M. (2018). Cyclic Fatigue Resistance of R-Pilot, HyFlex EDM and PathFile Nickel-Titanium Glide Path Files in Artificial Canals with Double (S-Shaped) Curvature. Int. Endod. J..

[74] Yılmaz K., Uslu G., Özyürek T. (2017). In Vitro Comparison of the Cyclic Fatigue Resistance of HyFlex EDM, One G, and ProGlider Nickel Titanium Glide Path Instruments in Single and Double Curvature Canals. Restor. Dent. Endod..

[75] Özyürek T., Uslu G., Gündoğar M., Yılmaz K., Grande N.M., Plotino G. (2018). Comparison of Cyclic Fatigue Resistance and Bending Properties of Two Reciprocating Nickel-Titanium Glide Path Files. Int. Endod. J..

[76] Topçuoğlu H.S., Topçuoğlu G., Kafdağ Ö., Arslan H. (2018). Cyclic Fatigue Resistance of New Reciprocating Glide Path Files in 45- and 60-Degree Curved Canals. Int. Endod. J..

[77] Serefoglu B., Kaval M.E., Micoogullari Kurt S., Çalişkan M.K. (2018). Cyclic Fatigue Resistance of Novel Glide Path Instruments with Different Alloy Properties and Kinematics. J. Endod..

[78] Keskin C., İnan U., Demiral M., Keleş A. (2018). Cyclic Fatigue Resistance of R-Pilot, WaveOne Gold Glider, and ProGlider Glide Path Instruments. Clin. Oral Investig..

[79] Yılmaz K., Uslu G., Gündoğar M., Özyürek T., Grande N.M., Plotino G. (2018). Cyclic Fatigue Resistances of Several Nickel-Titanium Glide Path Rotary and Reciprocating Instruments at Body Temperature. Int. Endod. J..

[80] Nishijo M., Ebihara A., Tokita D., Doi H., Hanawa T., Okiji T. (2018). Evaluation of Selected Mechanical Properties of NiTi Rotary Glide Path Files Manufactured from Controlled Memory Wires. Dent. Mater. J..

[81] Topçuoğlu H.S., Topçuoğlu G., Düzgün S. (2018). Resistance to Cyclic Fatigue of PathFile, ScoutRaCe and ProGlider Glide Path Files in an S-Shaped Canal. Int. Endod. J..

[82] Kırıcı D., Kuştarcı A. (2019). Cyclic Fatigue Resistance of the WaveOne Gold Glider, ProGlider, and the One G Glide Path Instruments in Double-Curvature Canals. Restor. Dent. Endod..

[83] Lee J.-Y., Kwak S.W., Ha J.-H., Abu-Tahun I.H., Kim H.-C. (2019). Mechanical Properties of Various Glide Path Preparation Nickel-Titanium Rotary Instruments. J. Endod..

[84] Oh S., Seo J.-Y., Lee J.-E., Kim H.-J., Jang J.-H., Chang S.W. (2022). Evaluation of Design, Mechanical Properties, and Torque/Force Generation of Heat-Treated NiTi Glide Path Instruments. BMC Oral Health.

[85] Martins J.N.R., Marques D., Vasconcelos I., Arantes-Oliveira S., Caramês J., Braz Fernandes F.M. (2022). Multimethod Assessment of the Cyclic Fatigue Strength of ProGlider, Edge Glide Path and R-Pilot Endodontic Instruments. Dent. J..

[86] Paleker F., van der Vyver P.J. (2017). Glide Path Enlargement of Mandibular Molar Canals by Using K-Files, the ProGlider File, and G-Files: A Comparative Study of the Preparation Times. J. Endod..

[87] D’Agostino A., Cantatore G. (2014). Glide-Path: Comparison between Manual Instruments, First Generation Rotary Instruments and M-Wire New Generation Rotary Instruments. G. Ital. Di Endod..

[88] Gambarini G., Galli M., Cicconetti A., Di Nardo D., Seracchiani M., Obino F.V., Miccoli G., Testarelli L. (2021). Operative Torque Analysis to Evaluate Cutting Efficiency of Two Nickel-Titanium Rotary Instruments for Glide Path: An In Vitro Comparison. J. Contemp. Dent. Pract..

[89] Han Y., Hou X.-M. (2021). Glide Path Enlargement of Curved Molar Canals Using HyFlex EDM Glide Path File versus PathFile: A Comparative Study of Preparation Time and Postoperative Pain. BMC Oral Health.

[90] Alcalde M.P., Duarte M.A.H., Calefi P.H.S., Cruz V.D.M., Vasconcelos B.C.D., Só M.V.R., Vivan R.R. (2021). Evaluation of Type of Kinematics on Glide Path Procedures and Torsional Fatigue Resistance after Preparation of Moderately Curved Canals. Braz. Oral Res..

[91] Vorster M., van der Vyver P.J., Paleker F. (2018). Influence of Glide Path Preparation on the Canal Shaping Times of WaveOne Gold in Curved Mandibular Molar Canals. J. Endod..

[92] Berutti E., Alovisi M., Pastorelli M.A., Chiandussi G., Scotti N., Pasqualini D. (2014). Energy Consumption of ProTaper Next X1 after Glide Path with PathFiles and ProGlider. J. Endod..

[93] Adıguzel M., Tufenkci P. (2018). Comparison of the Ability of Reciproc and Reciproc Blue Instruments to Reach the Full Working Length with or without Glide Path Preparation. Restor. Dent. Endod..

[94] Ramyadharshini T., Sherwood I.A., Vigneshwar V.S., Ernest Prince P., Vaanjay M. (2020). Influence of Glide Path Size and Operating Kinetics on Time to Reach Working Length and Fracture Resistance of Twisted File Adaptive and Endostar E3 Nickel-Titanium File Systems. Restor. Dent. Endod..

[95] Jena D., Bansal N., Batra D., Arora A., Gupta R., Dudulwar D.G. (2021). Assessment of Different File Systems for Working Time Based on Glide Path, Operating Kinetics, and the Fracture Resistance. J. Contemp. Dent. Pract..

[96] Kwak S.-W., Ha J.-H., Lee W., Kim S.-K., Kim H.-C. (2014). Buckling Resistance, Bending Stiffness, and Torsional Resistance of Various Instruments for Canal Exploration and Glide Path Preparation. Restor. Dent. Endod..

[97] Arias A., Singh R., Peters O.A. (2016). Differences in Torsional Performance of Single- and Multiple-Instrument Rotary Systems for Glide Path Preparation. Odontology.

[98] Al Raeesi D., Kwak S.W., Ha J.-H., Sulaiman S., El Abed R., Kim H.-C. (2018). Mechanical Properties of Glide Path Preparation Instruments with Different Pitch Lengths. J. Endod..

[99] Gavini G., Akisue E., Kawakami D.A.S., Caldeira C.L., Candeiro G.T.D.M., Vivan R.R., Calefi P.H.S., Alcalde M.P., Duarte M.A.H. (2021). Optimum Glide Path Motion Is Safer than Continuous Rotation of Files in Glide Path Preparation. Aust. Endod. J..

[100] Santos C.B., Simões-Carvalho M., Perez R., Vieira V.T.L., Antunes H.S., Cavalcante D.F., De-Deus G., Silva E.J.N.L. (2019). Torsional Fatigue Resistance of R-Pilot and WaveOne Gold Glider NiTi Glide Path Reciprocating Systems. Int. Endod. J..

[101] Lopes W.S.P., Vieira V.T.L., Silva E.J.N.L., Silva M.C.D., Alves F.R.F., Lopes H.P., Pires F.R. (2020). Bending, Buckling and Torsional Resistance of Rotary and Reciprocating Glide Path Instruments. Int. Endod. J..

[102] Sivas Yilmaz Ö., Keskin C., Aydemir H. (2021). Comparison of the Torsional Resistance of 4 Different Glide Path Instruments. J. Endod..

[103] İnan U., Keskin C. (2019). Torsional Resistance of ProGlider, Hyflex EDM, and One G Glide Path Instruments. J. Endod..

[104] Arias A., de Vasconcelos R.A., Hernández A., Peters O.A. (2017). Torsional Performance of ProTaper Gold Rotary Instruments during Shaping of Small Root Canals after 2 Different Glide Path Preparations. J. Endod..

[105] Abu-Tahun I.H., Kwak S.W., Ha J.-H., Sigurdsson A., Kayahan M.B., Kim H.-C. (2019). Effective Establishment of Glide-Path to Reduce Torsional Stress during Nickel-Titanium Rotary Instrumentation. Materials.

[106] Dagna A., El Abed R., Hussain S., Abu-Tahun I.H., Visai L., Bertoglio F., Bosco F., Beltrami R., Poggio C., Kim H.-C. (2017). Comparison of Apical Extrusion of Intracanal Bacteria by Various Glide-Path Establishing Systems: An in Vitro Study. Restor. Dent. Endod..

[107] Low N., Zhen Jie S., Bhatia S., Davamani F., Nagendrababu V. (2021). Comparison of Apical Extrusion of Bacteria After Glide Path Preparation Between Manual K File, One G Rotary, and WaveOne Gold Glider Reciprocation Preparations. Eur. Endod. J..

[108] Ha J.-H., Kim S.K., Kwak S.W., El Abed R., Bae Y.C., Kim H.-C. (2016). Debris Extrusion by Glide-Path Establishing Endodontic Instruments with Different Geometries. J. Dent. Sci..

[109] Keskin C., Sivas Yilmaz Ö., Inan U. (2020). Apically Extruded Debris Produced during Glide Path Preparation Using R-Pilot, WaveOne Gold Glider and ProGlider in Curved Root Canals. Aust. Endod. J..

[110] Topçuoğlu H.S., Düzgün S., Akpek F., Topçuoğlu G., Aktı A. (2016). Influence of a Glide Path on Apical Extrusion of Debris during Canal Preparation Using Single-File Systems in Curved Canals. Int. Endod. J..

[111] Kırıcı D., Koç S., Kuştarcı A. (2020). Effects of Different Glide Path Techniques on the Amount of Extruded Debris and Preparation Times during Root Canal Preparation. J. Dent. Res. Dent. Clin. Dent. Prospect..

[112] Pawar A.M., Pawar M., Kfir A., Thakur B., Mutha P., Banga K.S. (2017). Effect of Glide Path Preparation on Apical Extrusion of Debris in Root Canals Instrumented with Three Single-File Systems: An Ex Vivo Comparative Study. J. Conserv. Dent..

[113] Thu M., Ebihara A., Maki K., Kimura S., Kyaw M.-S., Kasuga Y., Nishijo M., Okiji T. (2023). Dynamic Torque and Screw-in Force of Four Different Glide Path Instruments Assessed in Simulated Single- and Double-Curved Canals: An in Vitro Study. J. Dent. Sci..

[114] Kwak S.W., Ha J.-H., Shen Y., Haapasalo M., Kim H.-C. (2022). Comparison of the Effects from Coronal Pre-Flaring and Glide-Path Preparation on Torque Generation during Root Canal Shaping Procedure. Aust. Endod. J..

[115] Kwak S.W., Ha J.-H., Cheung G.S.-P., Kim H.-C., Kim S.K. (2018). Effect of the Glide Path Establishment on the Torque Generation to the Files during Instrumentation: An In Vitro Measurement. J. Endod..

[116] Topçuoğlu H.S., Düzgün S., Akpek F., Topçuoğlu G. (2016). Effect of Glide Path and Apical Preparation Size on the Incidence of Apical Crack during the Canal Preparation Using Reciproc, WaveOne, and ProTaper Next Systems in Curved Root Canals: A Stereomicroscope Study. Scanning.

[117] Aktemur Türker S., Uzunoğlu E. (2015). Influence of a Glide Path on the Dentinal Crack Formation of ProTaper Next System. Restor. Dent. Endod..

[118] Saber S.E.D.M., Schäfer E. (2016). Incidence of Dentinal Defects after Preparation of Severely Curved Root Canals Using the Reciproc Single-File System with and without Prior Creation of a Glide Path. Int. Endod. J..

[119] Bürklein S., Werneke M., Schäfer E. (2018). Impact of Glide Path Preparation on the Incidence of Dentinal Defects after Preparation of Severely Curved Root Canals. Quintessence Int..

[120] Jonker C.H., Van der Vyver P.J., De Wet F.A. (2014). The Influence of Glide Path Preparation on the Failure Rate of WaveOne Reciprocating Instruments. South Afr. Dent. J..

[121] Ehrhardt I.C., Zuolo M.L., Cunha R.S., De Martin A.S., Kherlakian D., de Carvalho M.C.C., Bueno C.E.D.S. (2012). Assessment of the Separation Incidence of Mtwo Files Used with Preflaring: Prospective Clinical Study. J. Endod..

[122] Barbosa Machado A.L., Machado A.G., Silveira Bueno C.E. (2019). Surface Changes of WaveOne^TM^ and Reciproc^®^ Instruments after Using Three Times for Preparation of Simulated Curved Canals with and without Glide Path. Iran. Endod. J..

[123] Türker S.A., Sağlam B.C., Koçak M.M., Koçak S. (2014). The Effect of Glide Path on the Surface Quality of New and Used Rotary and Reciprocating Single Files: OneShape versus WaveOne. Scanning.

[124] Pasqualini D., Mollo L., Scotti N., Cantatore G., Castellucci A., Migliaretti G., Berutti E. (2012). Postoperative Pain after Manual and Mechanical Glide Path: A Randomized Clinical Trial. J. Endod..

[125] Keskin C., Sivas Yilmaz Ö., Inan U., Özdemir Ö. (2019). Postoperative Pain after Glide Path Preparation Using Manual, Reciprocating and Continuous Rotary Instruments: A Randomized Clinical Trial. Int. Endod. J..

[126] Adıgüzel M., Yılmaz K., Tüfenkçi P. (2019). Comparison of Postoperative Pain Intensity after Using Reciprocating and Continuous Rotary Glide Path Systems: A Randomized Clinical Trial. Restor. Dent. Endod..

[127] Tufenkci P., Adiguzel M., Yilmaz K. (2019). Intraoperative Pain During Glide Path Creation with the Use of a Rotary or Reciprocating System. Cumhur. Dent. J..

[128] De-Deus G., Belladonna F.G., Souza E.M., de Oliveira Alves V., Silva E.J.N.L., Rodrigues E., Versiani M.A., da Silveira Bueno C.E. (2016). Scouting Ability of 4 Pathfinding Instruments in Moderately Curved Molar Canals. J. Endod..

[129] Pedullà E., Leanza G., La Rosa G.R.M., Gueli A.M., Pasquale S., Plotino G., Rapisarda E. (2020). Cutting Efficiency of Conventional and Heat-Treated Nickel-Titanium Rotary or Reciprocating Glide Path Instruments. Int. Endod. J..

[130] Pereira R.P., Alcalde M.P., Duarte M.A.H., Vivan R.R., Bueno C.E.S., Duque J.A., Calefi P.H.S., Bramante C.M. (2021). A Laboratory Study of the Scouting Ability of Two Reciprocating Glide Path Instruments in Mesial Root Canals of Extracted Mandibular Molars. Int. Endod. J..

[131] Campos D.S., Rodrigues E.A., Bueno C.E.D.S., Fontana C.E., da Silva E.J.N.L., de Lima C.O., De Martin A.S. (2021). The Ability of Reciprocating Glide Path Instruments to Reach the Full Root Canal Working Length. Aust. Endod. J..

